# Is sedentary behaviour just physical inactivity by another name?

**DOI:** 10.1186/s12966-017-0601-0

**Published:** 2017-10-23

**Authors:** Hidde P. van der Ploeg, Melvyn Hillsdon

**Affiliations:** 1 Department of Public and Occupational Health, Amsterdam Public Health Research Institute, VU University Medical Centre, van der Boechorststraat 7, NL-1081, BT Amsterdam, the Netherlands; 2 Sydney School of Public Health, University of Sydney, Camperdown, Australia; 3 College of Life and Environmental Sciences, University of Exeter, Exeter, UK

**Keywords:** Sedentary behaviour, Physical activity, Public health

## Abstract

The relationship between sedentary behaviour and physical activity and their role in the development of health conditions is an ongoing topic of research. This debate paper presents arguments in favour and against the statement: *“Is sedentary behaviour just physical inactivity by another name?”* The paper finishes with recommendations for future research in the field of sedentary behaviour, physical activity and public health.

## Background

Following an ever growing body of scientific literature, sedentary behaviour has received considerable public and media attention. Sedentary behaviour is defined as any waking behaviour characterized by an energy expenditure ≤1.5 METs while in a sitting, reclining or lying posture [1, 2]. Within the scientific community there is an ongoing discussion on how sedentary behaviour is related to physical activity, and whether sedentary behaviour is an independent risk factor for cardio-metabolic health. At the 2016 HEPA Europe conference in Belfast (Northern Ireland), this was the subject of a scientific debate. The current paper summarizes both the arguments in favour and against the general statement of that debate: *“Is sedentary behaviour just physical inactivity by another name?”*

## Sedentary behaviour is not the same as physical inactivity (Hidde van der Ploeg)

The answer to the question if ‘sedentary behaviour is just physical inactivity by another name’ is of course dependent on their respective definitions. With the definition of sedentary behaviour already formulated in the introduction above, this leaves us with the definition of physical inactivity. In line with the widely adopted Sedentary Behaviour Research Network definitions, most research studies these days use the term inactive to describe people who are performing insufficient amounts of moderate- and vigorous-intensity activity (MVPA), i.e. not meeting specified physical activity guidelines [1, 2]. The WHO global recommendations on physical activity for health state that “Adults aged 18–64 years should do at least 150 min of moderate-intensity aerobic physical activity throughout the week, or do at least 75 min of vigorous-intensity aerobic physical activity throughout the week, or an equivalent combination of moderate- and vigorous-intensity activity (MVPA) [3].” WHO defined moderate-intensity physical activity as any activity with a MET value between 3 and 5.9 and vigorous-intensity physical activity as ≥6 MET.

Following this definition of inactivity, it becomes clear that inactivity and sedentary behaviour are different constructs. Here is why. Nicole drives to work in the morning, sits behind her computer and in meetings all day at work, drives back home and spends most of the evening watching television with her family. That was a highly sedentary day for Nicole. Nevertheless, it was not an inactive day, as she also went running for 30 min, which puts her on target to meet the WHO physical activity recommendation. So Nicole was highly sedentary and physically active on the same day. The opposite is also possible. For example, people with a standing occupation (e.g. hairdresser, shop sales person) will get little sedentary time during their day but might not do any MVPA. Hence, they are inactive but not sedentary. In other words, sedentary behaviour and inactivity are indeed two distinct constructs, and sedentary behaviour and MVPA are not the inverse of each other.

### Energy expenditure spectrum

Nevertheless, sedentary behaviour and MVPA are part of the same energy expenditure spectrum. To meet the WHO physical activity recommendation only around 2% of our waking time needs to be spent in MVPA [3]. The remaining 98% of our waking time is by definition spent in sedentary behaviour and light intensity activity. Light intensity activity is defined as any activity with an energy expenditure between 1.5–3 MET, which includes both static (e.g. standing) and ambulatory activities. Sedentary behaviour and light intensity physical activity are highly correlated and almost the inverse of each other.

In addition to meeting the MVPA public health recommendations, it is that balance between sedentary behaviour and light intensity physical activity that also seems to be important for public health. In other words, it might be important to consider all behaviours across the energy expenditure spectrum, and not just the least frequently practiced top end (MVPA), even though (M)VPA might have the biggest health gains. This is reflected in the inclusion of sedentary behaviour recommendations in several recently updated national physical activity guidelines [4–8]. The energy expenditure spectrum argument is also responsible for the often included ‘more is better’ message in recent physical activity recommendations. It is important to note that such recommendations provide the public with an easy to understand physical activity target, which does not represent a dichotomous cut off between non-beneficial and highly beneficial MVPA levels.

### Evidence from prospective cohort studies

In 2012, two large prospective cohort studies (*N* > 200,000) from Australia and the US reported an association between mortality and total sitting time (as proxy for sedentary time) next to the well-documented association between mortality and physical inactivity [9, 10]. These two cohort studies and a subsequent meta-analysis including a total of six studies suggested that sitting time in itself was not hazardous but only in higher volumes [11]. The meta-analysis showed that around 7–8 h of self-reported sitting per day, the hazard ratios for all-cause mortality started to increase significantly [11].This suggested that sitting was only problematic in high doses. Hence, it is important to realize that sitting is a natural behaviour, which should not be demonized or completely banned.

In the summer of 2016, the second special issue on physical activity in the Lancet included a harmonised meta-analysis of sitting time, physical activity and mortality (i.e. all-cause, cardiovascular and cancer mortality) [12]. The meta-analysis included well over a million people who were apparently healthy at baseline and the authors concluded that high levels of moderate intensity physical activity seem to eliminate the increased risk of death associated with high sitting time. In line with the earlier meta-analysis, their results also suggested that mortality hazard ratios (i.e. all-cause and cardiovascular mortality) increase significantly from ~8 h of sitting per day onwards. However, the results in the least active group (≤2.5 MET-h/wk. of MVPA) seemed to suggest there might be more of a dose-response relationship in this group and lower levels of sitting time might already be considered hazardous. An important implication of this is that the inactive and high sedentary segment of the population is at the highest risk and needs special attention in public health efforts.

The good news in the Lancet meta-analysis was that MVPA attenuated and even eliminated the detrimental association between sitting and mortality. But how much MVPA is needed to achieve this? The detrimental effects of sitting were eliminated in the highest MVPA group, who did ≥35.5 MET-h/wk. of MVPA. The equivalent of meeting the WHO physical activity recommendation is 7.5 MET-h/wk. [3], which means that people would have to do at least 4–5 times more than the WHO physical activity recommendation to offset the detrimental effects of sitting too much. From a public health point of view doing 4–5 times as much as the WHO physical activity recommendation seems feasible for only a small portion of the population as many already struggle to meet the WHO recommendation in itself. It has been estimated that 31.1% of adults worldwide do not meet physical activity recommendations [13], although such estimates are highly dependent on study and assessment methods [14]. Nevertheless, it is important to note that ‘at least 4-5 times more than the WHO physical activity recommendation’ is a rough estimate. There was substantial heterogeneity in the included cohort studies in the meta-analysis for both the MVPA assessment methods (and associated measurement bias) and in the consequently reported absolute MVPA levels. For this reason the authors had to work with MVPA quartiles for each individual study. These were then combined for all studies, leading to substantial heterogeneity of MVPA levels in the different quartiles.

Better standardized or objective assessment of MVPA in the included cohort studies would have led to a better estimate of the role of MVPA. Unfortunately, little objective physical activity and sedentary behaviour data is available in prospective cohort studies to date. However, the available objective data reports findings similar to that of the Lancet meta-analysis, which included mostly self-reported data. The accelerometer data from NHANES (*n* = 4840) shows that increasing light-intensity activity and reducing sedentary time are important to reduce the risk of mortality, particularly for inactive adults [15].

### Limitations of prospective cohort studies

Prospective cohort studies are of course not without methodological limitations. Especially, the potential for reverse causality and residual confounding warrant special attention. Reverse causality has been investigated extensively in the discussed cohort studies, namely through the exclusion of people with pre-existing disease from the analyses, adjusting for health status, and through sensitivity analyses excluding people who died in the first few years of follow up. In order to minimize residual confounding, adjustment for confounding in the different prospective cohort studies has been rather extensive (often including confounders such as socioeconomic status, health status, smoking, diet and BMI). Although reverse causality and residual confounding can never be ruled out in prospective cohort studies, the association between sitting time and mortality (all-cause and cardiovascular) has been rather consistent when using the above described statistical adjustment methods and sensitivity analyses. Finally, to date no clear candidate residual confounder has been identified that has never been adjusted for in such cohort studies.

Another limitation is the lack of available prospective cohort data around the potential role of different types and patterns of sedentary behaviour. It is possible that different patterns and types of sedentary behaviour present somewhat different health risks. For example, it is possible that reclining on the couch has different health implications than sitting in your office chair working hard behind your computer, and a small difference in energy expenditure between sitting while watching TV and sitting while typing has been reported [16]. Furthermore, a review of experimental lab studies has suggested prolonged sitting might be more detrimental for metabolic health than the same amount of sitting in a more broken up pattern [17]. This seems to suggest that it is not only the total volume of sitting that matters for health but also the pattern in which sitting time is accumulated.

### Reducing sedentary behaviour

Although MVPA appears to attenuate the risks of sedentary behaviour, unrealistically large volumes of MVPA appear to be needed from a public health point of view to completely eliminate the risks of sedentary behaviour. Furthermore, MVPA can only play a small role in the reduction of large volumes of sedentary time. To get a person who is sedentary for 10 h/d and does no MVPA, to meet the WHO physical activity recommendations is a great accomplishment from an MVPA and health point of view, but is also challenging from a behaviour change point of view. Furthermore, the impact of increasing MVPA on the volume of sedentary time is limited, as a substantial increase of 0.5 h/d in MVPA results in a relatevily small reduction in sedentary time to 9.5 h/d.

Hence, when trying to reduce sedentary behaviour, not only MVPA should be targeted but also the light intensity part of the energy expenditure spectrum. As discussed above the light intensity part of the energy expenditure spectrum includes static (e.g. standing) and ambulatory activities. The question is if any type of light intensity activity (and especially standing) can have health benefits when replacing sedentary behaviour. Prospective cohort studies have reported inverse associations between standing time and all-cause mortality [18, 19]. However, little is known about the measurement properties of methods used to assess standing time in these studies and they might reflect upright time rather than standing still time.

When looking more closely at standing, there is only a small difference in energy expenditure with sitting [16, 20]. For example, the physical activity compendium rates ‘sitting, talking in person, on the phone, computer, or text messaging, light effort’ as 1.5 MET and ‘standing, talking in person, on the phone, computer, or text messaging, light effort’ as 1.8 MET [20]. However, substituting a large volume of sitting with standing throughout the day would still lead to a substantial difference in energy expenditure over the whole day. Furthermore, the difference in leg muscle activity between sitting and standing might also have different effects on cardio-metabolic health. A review of lab based studies suggests that replacing sitting with light intensity activity and even standing may be a stimulus sufficient enough to induce acute favourable changes in postprandial metabolic parameters [17].

Hence, more evidence is needed around the health benefits of replacing sitting with standing. Sit-stand desk pilot intervention studies have shown substantial reductions in sitting time due to almost exclusively increases in standing time [21]. Full blown randomized controlled trials evaluating such interventions might be able to provide more definite evidence of the cardio-metabolic health effects of replacing sitting with standing time in sedentary individuals.

Finally, it must be noted that prolonged standing has its own health risks in the form of musculoskeletal problems such as low back pain [22]. Hence, standing all day also has health risks and a good public health message might be to alternate sitting, standing and other light, moderate, and vigorous physical activity throughout the day. This message also complements the occupational health recommendation to include variation in posture during work to reduce the risk of musculoskeletal complaints [23].

## Conclusion

Sedentary behaviour is not physical inactivity by a different name, but both should be targeted simultaneously in public health strategies. Even though many questions still remain, the accumulated epidemiological evidence suggests high volumes of sedentary time have a negative impact on especially cardio-metabolic health. Substantial amounts of MVPA do not only have clear health benefits but also attenuate the health risks of high volumes of sedentary time. The inactive and highly sedentary segment of the population is at greatest risk of poor health and needs special attention in public health efforts. From a public health point of view, MVPA by itself is insufficient to eliminate the risks of sedentary behaviour. A more integral public health recommendation might be to find a healthy balance between sitting, standing and other light intensity physical activity, and MVPA throughout the day.

## Sedentary behaviour is just physical inactivity by another name (Melvyn Hillsdon)

It has been claimed that sedentary behaviour (the posture of sitting and an energy expenditure ≤1.5 METs) [2] is an independent risk factor for poor health. The claim is based on a large number of studies that have reported positive associations between sedentary behaviour and risk of cardio-metabolic diseases and all-cause mortality [11, 24, 25]. Such studies have seemingly been deemed sufficient for some countries to incorporate guidelines for limiting sedentary behaviour alongside their guidelines for physical activity [4, 6]. The publication of government guidelines on sedentary behaviour would be entirely appropriate if the underpinning research was robust, but if it is not robust then the guidelines could be counter-productive and potentially detrimental to public health. It is therefore crucial that we examine the robustness of the evidence in order to substantiate, or otherwise, the appropriateness of these guidelines.

Although there is a large body of evidence reporting associations between time spent in sedentary behaviours and poor health, there is an increasing body of evidence reporting no association [26–30]. It may also be the case that there are more unpublished studies that have found no association due to the tendency to view null findings as less interesting and and less publishable compared to the enthusiasm for studies with positive associations. One of the main reasons for the equivocal findings is likely due to differences in methodologies between those that do and do not find an association. This article will highlight three primary methodological limitations of observational studies – confounding, causality and measurement error - and demonstrate the impact they have in studies that do and do not address them.

## Inadequate adjustment for confounding

The claim that sitting is an ‘independent’ risk factor implies that there is something about the posture of sitting that is detrimental to health and that the associated risk is not simply reflecting very low levels of physical activity or other health-effecting behaviours that correlate with sitting. If the true risk factor for future health is sedentary behaviour, and not physical inactivity, then associations between sedentary behaviour and health outcomes need to control for total physical activity. That is “any bodily movement produced by skeletal muscles that results in energy expenditure” with physical inactivity the absence of this [31]. It is common in studies of the association between sedentary behaviour and mortality to assert that associations remain after adjustment for physical activity [11]. Closer inspection of these studies reveals that only certain types of physical activity have been adjusted for rather than total physical activity. In most cases only time spent in moderate-and-vigorous intensity physical activity (MVPA: ≥3 METs) has been statistically adjusted for. Given MVPA only accounts for approximately 10% of total physical activity [32], depending on definition, this leaves the majority of physical activity, at intensities < MVPA, unaccounted for even though time spent in light intensity physical activity (1.5–2.9 METs) is associated with improved health outcomes [33] and is, crucially, strongly inversely co-correlated with time spent sedentary - *r* = −0.99 to −0.96 [34]. If no adjustment is made for light physical activity then it is difficult to determine whether the association with poor health is due to more time sedentary or less time in light activity. On the limited number of occasions that total physical activity has been controlled for, both cross-sectional and longitudinal associations between sedentary behaviour and health outcomes are attenuated toward the null [35, 36]. Furthermore, only controlling for time spent above the threshold of moderate intensity assumes that health benefits of physical activity at 3.1 METs is the same as 12 METs which seems highly unlikely [37]. If controlling for total volume of physical activity attenuates associations between time spent sedentary and health outcomes to the null then it is most likely that sedentary behaviour is simply displacing physical activity and it is the absence of physical activity that is the underlying exposure not sedentary behaviour. The counter argument authors often give for not controlling for total physical activity is that because there are a finite number of minutes in each day, doing so would lead to issues of complete collinearity (*r* = 1.00) between time spent sedentary and total time in physical activity (not sedentary) [38]. The problem of collinearity is only true if derived measures of physical activity at different intensities are expressed in units of time. If volume of work is the derived measure (counts per minute or milli-g- seconds based from accelerometers or kcal/min, MET-mins per week estimated from self-reports) then the problem of collinearity is alleviated as there is no finite upper limit.

Another fundamental limitation to the claim that sitting is an independent risk factor is that not all types of sitting have been shown to increase risk [36, 39]. In fact, according to a systematic review, one of the most common forms of sitting, occupational sitting, does not appear to increase risk [40]. This would suggest that the forms of sitting that have been shown to increase risk may be due to confounding factors or a difference in how the volume of sitting was accumulated. For example, TV viewing is one type of sitting that has been consistently reported to be associated with health outcomes [39, 41] and yet that form of sitting is known to be associated with other behaviours such as snacking [42, 43].

Sitting for different purposes are likely to have specific health related correlates that may confound associations with health. For example, occupational social class is likely to be related to occupational sitting, car ownership and income with time sitting in cars and unhealthy snacking whilst watching TV. Statistical adjustment for socioeconomic correlates specific to the type of sitting are rarely, if ever undertaken. This is an important limitation as more time spent watching TV is associated with low socioeconomic position [44] and lower socioeconomic position is a risk factor for negative health outcomes [45]. Studies often do statistically control for some aspects of dietary behaviour such as alcohol consumption, fat consumption and energy intake. Although general measures of unhealthy diet are correlated with TV viewing time this is quite different from measuring the food consumed during the period when viewing TV [46]. Adjustment for general dietary habits may miss the co-occurrence with TV viewing. More momentary assessments of diet and sedentary behaviour may be required to better disentangle this association [47]. For example, sitting to watch TV immediately after a large, calorie dense meal may be more detrimental than sitting before a meal or sitting at work an hour after breakfast and a short walk on the way to work. Fully adjusting for confounders might be expected to re-align associations between time spent watching TV and health with those reported with occupational sitting time and health.

An alternative explanation for the differences in risks reported between health and TV viewing and other types of sedentary behaviour is that the pattern of sitting is more prolonged while watching TV and it is the long periods of uninterrupted sitting that confer the greatest risk to health rather than total volume of sitting [48]. Evidence from experiments of breaks in prolonged sitting are normally cited to substantiate this hypothesis [49]. A meta-analysis of studies examining the effects of, or relationship between, breaks in sedentary behaviour and cardio-metabolic health reported that breaking up continuous periods of sedentary behaviour with short bouts of light intensity physical activity improved blood glucose levels compared to continuous sitting, whereas bouts of standing did not [50]. Rather than providing evidence of the detrimental effects of uninterrupted periods of sedentary behaviour these results indicate that short bouts of light intensity physical activity are beneficial in reducing blood glucose levels. Even if it was the case that only prolonged bouts of continuous sitting were detrimental this would only be of public health significance if it was prevalent in the general population rather than through manipulation/enforcement in the laboratory. Objective data on patterns of sitting and transitions to upright postures suggest that long uninterrupted periods of sitting are not common. In a study of office workers 69% of weekday and 68% of weekend sitting bouts lasted between 0 and 10 min and less than 5% of total sitting bouts were longer than 30 min. Further, the average number of transitions per hour was still above 2 even during the evening when people were more likely to be watching TV [51].

## Assumptions about causality

Another important limitation in observational studies is their inability to determine cause and effect, often because factors upstream on the causal pathway are not included in the analysis. This limitation could certainly pertain to the claim that sedentary behaviour leads to poor health.

Antecedents of sitting may include cardiorespiratory fitness (CRF) and Body Mass Index (BMI). Cross sectional and longitudinal associations between sitting time and cardio-metabolic risk factors have been shown to be attenuated when CRF is controlled for [52, 53] and in a longitudinal study of middle-aged adults, change in CRF mediated the association between sedentary behaviour and a cardio-metabolic risk score whereby the association was only present in those with reduced fitness [54]. It is possible therefore that low fit adults gradually become less physically active which further reduces CRF and leads to an increased risk of premature mortality.

The evidence also suggests that the link between sitting and obesity can be explained by reverse causality – obesity leads to more sitting but more sitting does not lead to obesity. Prospective associations between sedentary behaviour and risk of developing obesity are weak and inconsistent yet those between obesity and risk of becoming inactive/sedentary are consistently stronger [55]. Studies that fail to take account of these and other potential antecedents could make incorrect inferences regarding causality.

## Measurement error

Determining that sedentary behaviour is detrimental to health and that the associated risk is not simply reflecting a lack of physical activity requires precise measurement of both behaviours and their correlates to avoid misclassification. There are many measures, both self-report and objective, of both behaviours. Most of these do not have published validity and reliability data from studies in free-living settings, especially measures of sedentary behaviour. Some authors report the time spent below a specific level of acceleration, derived from wrist or waist worn accelerometers, as a measure of the volume of sedentary behaviour [56]. This is misleading as this is simply a measure of inactivity on the opposite end of the same scale used to estimate physical activity. Although accelerometers are available that can discriminate between different postures and physical activity, they are not yet widely used in population studies. Because patterns of both physical activity and sitting, along with their relationship with other health behaviours, are potentially as important as the volume of each behaviour [57, 58], it is insufficient to simply report weekly or average daily volumes of each behaviour. A better understanding of the temporal sequence of bouts of physical activity (of varying intensities and durations) would provide a more precise estimate of the attenuating effect of both the volume and pattern of all physical activities on the association between sedentary behaviour and health. Failure to statistically adjust for the pattern of physical activity, as well as the total volume, may still leave residual confounding that could be misinterpreted as an ‘independent association’.

## Conclusion

The methodological limitations highlighted above also apply to many studies of the association between physical activity and health and determining when the evidence is sufficient for public health action requires a balanced approach. However, the equivocal results from observational studies and the substantial methodological limitations highlighted in this paper do not support the claim that sedentary behaviour leads to poor health and the subsequent guidelines cannot currently be substantiated. What we can safely say is that not moving (physical inactivity) is bad for you but how you stay still probably doesn’t matter.

## Joint recommendations for future research

-Studies are advised to incorporate high quality assessment methods for physical activity and sedentary behaviour, ideally using objective assessment methods but preferably also including information on domains (e.g. leisure, occupational, etc.), types (e.g. sitting on couch watching TV) and context of behaviour.

-Studies are advised to take account of other factors that may confound the association between specific sedentary behaviours and health outcomes.

-Study the role of different domains and types of sedentary behaviour with regard to health outcomes.

-Study the roles of total volume and patterns (bouts and breaks) of both physical activity (at all intensities) and sedentary behaviour with regard to health outcomes.

-Study the health effects of replacing sedentary behaviour with standing or light intensity physical activities.

-Study the dose-response relationships between physical activity, sedentary behaviour and health outcomes.

-Study the underlying physiological mechanisms of the relationship between sedentary behaviour, physical activity and health outcomes.

-Conduct intervention studies to establish the direction of causality between sedentary behaviour, physical activity and health outcomes.

## Additional recommendations

### Hidde van der Ploeg

-Studies are advised to use assessment methods and analysis techniques that take into account the energy expenditure spectrum as a continuous measure of intensity level, rather than grouping physical activity into categorical intensity groups (light, moderate, vigorous), as this can provide more accurate estimates of the relationship with health.

-Studies should avoid over-adjustment for light intensity activity when studying the relationship between sedentary behaviour and health, as light intensity activity is by definition almost the inverse of sedentary behaviour.

### Melvyn Hillsdon

-Studies testing the independent association between sedentary behaviour and health are advised to adjust for total volume of physical activity (in mg or counts, not minutes), not just time spent in MVPA. Otherwise the resulting association may simply be reflecting the beneficial effects of doing more light intensity activity, not the detrimental effect of more time spent sedentary.
